# Real-life helping behaviours in North America: A genome-wide association approach

**DOI:** 10.1371/journal.pone.0190950

**Published:** 2018-01-11

**Authors:** Georg Primes, Martin Fieder

**Affiliations:** Department of Anthropology, University of Vienna, Vienna, Austria; Universita degli Studi di Roma Tor Vergata, ITALY

## Abstract

In humans, prosocial behaviour is essential for social functioning. Twin studies suggest this distinct human trait to be partly hardwired. In the last decade research on the genetics of prosocial behaviour focused on neurotransmitters and neuropeptides, such as oxytocin, dopamine, and their respective pathways. Recent trends towards large scale medical studies targeting the genetic basis of complex diseases such as Alzheimer’s disease and schizophrenia pave the way for new directions also in behavioural genetics.

Based on data from 10,713 participants of the American Health and Retirement Study we estimated heritability of helping behaviour–its total variance explained by 1.2 million single nucleotide polymorphisms–to be 11%. Both, fixed models and mixed linear models identified rs11697300, an intergene variant on chromosome 20, as a candidate variant moderating this particular helping behaviour. We assume that this so far undescribed area is worth further investigation in association with human prosocial behaviour.

## Introduction

Prosocial behaviour–voluntary behaviour intended to benefit others [1]–is essential for social functioning in humans, who, next to eusocial insects, form the largest cooperative living groups on Earth. Extensive research has been conducted focusing on individual differences in this multifaceted trait that covers concepts such as helping, cooperation, altruism, and empathy [2–4]

Ever since Hamilton [5] the evolution of social behaviour on a species level has been discussed in terms of genetics. Unsurprisingly, the traditional twin study approach suggests a partial hardwiring of human prosocial behaviour. Its heritability is typically estimated to be between 10 and 60%, increasing with age and varying with the respective concept of prosocial behaviour under investigation [6–9].

On the individual level, however, we are only just beginning to understand the genetic influences on human (pro)social behaviour. Research on the regulatory effects of neuropeptides such as oxytocin and vasopressin on social cognition and behaviour [10,11] and the search for their genetic basis have produced several candidate genes. These include the oxytocin receptor gene (OXTR), the argenine vasopressine receptor 1A (AVPR1A) as well as others involved in the dopamine and serotonin pathway of receptors (DRD4, 5-HTR), in synthesis and degradation (COMT, MAOA), and in transportation (DAT, SERT). Studies focusing on these candidate genes found associations with social cognitive functioning, complex medical conditions, as well as social behaviour [12–17].

The predominant method in investigating the genetic basis of prosocial behaviour and decision-making is the application of incentivized laboratory-based experiments derived from the field of experimental economics. These complement behavioural genetics approaches [18–22]. All the commonly employed games in behavioural economics experiments (e.g. Dictator Game, Ultimatum Game, Trust Game, Public Goods Game) are easily adaptable and are increasingly being combined with brain imaging techniques to generate insights into the neurobiological structure of economic decision making [23], for example. Beyond this modularity, the approach provides researchers with experimental control by allowing for controlled variation of a variable while keeping all other conditions constant. This both facilitates interpretation of results and simplifies study replication.

Nonetheless, there are several drawbacks to this approach, varying in their severity with the field of application. The sample size of laboratory-based experiments is often small, limiting the generalizability of the results [24]. The trade-off between internal validity in the laboratory and external validity is a genuine, broadly discussed problem [25]. Increasing the sample size creates costly and time-consuming logistics to set up the study. This is especially true when researchers combine standard games with brain imaging techniques and behavioural genetics approaches. Consequently, the latter commonly employ a target gene approach that allows only a small number of variations to be analysed.

Today, the increasing number of predominantly medical studies provides a vast collection of genetic data of large study samples. Their aim is to reveal genetic influences on complex diseases such as Alzheimer’s disease, breast cancer, and schizophrenia using genome-wide association approaches [26–28].

These studies are often designed as longitudinal studies to keep track of their participants over a longer period of time (Wisconsin Longitudinal Study http://www.ssc.wisc.edu/wlsresearch/, Health and Retirement Study http://hrsonline.isr.umich.edu/index.php, Avon Longitudinal Study of Parents and Children http://www.bristol.ac.uk/alspac/). The study teams also collect comprehensive phenotypic data beyond basic demographic information and medical condition. Therefore, these data sets provide an excellent opportunity to investigate genetic influences on 'every day' prosocial behaviour beyond strictly controlled laboratory-based experiments and on a much larger sample base. Simultaneously, recent progress in estimating heritability from whole genome sequence data [29] enable heritability research beyond the traditional twin study design.

To date, genome-wide association studies (GWAS) have not been used very frequently to identify the genomic basis of behavioural traits, besides the GWAS used in mental diseases research. Although GWAS have historically only explained a small proportion of the variance in a variety of complex traits being studied, they are well suited to detect unknown causal variants associated with a trait as in contrast to candidate gene tests GWAS are hypothesis free. They therefore offer the opportunity to gain completely new insights into the genetic basis of behaviour. In addition, large study data sets of unrelated individuals allow for an estimation of genome-wide variance explained which due to the availability of common causal variants usually present underestimates. A typical problem of GWAS is their limited potential to describe biological mechanisms on basis of GWAS results. Gene set analysis addresses this issue and uses GWAS results which describe a limited number of significantly associated SNP’s with a trait to estimate associations between the trait and entire gene sets known for their specific biological functions [30]. GWAS results also constitute the basis of the estimation of genetic correlations. This investigation of association between complex traits and diseases is especially relevant in gathering etiological insights in causal relationships [31].

All these points taken together, large study data sets provide a promising basis to explore new directions in behavioural genetics.

The goal of this study is to demonstrate new ways of exploring and investigating the genetic basis of (pro)social behaviour and decision making using established methods from medical/complex disease research. Not unlike complex diseases the genetic basis of a certain human behaviour is complex and heavily interdependent on various influence factors. However, unlike at least some complex diseases human prosocial behaviour is much more difficult to measure, quantify and describe compared to diseases and conditions with specified measurable symptoms.

This leads to the probably single most important limitation of the study presented here: the phenotypic representation of human prosocial behaviour by self-reported helping behaviour. The amount of time a person spends in order to help out his/her family, friends and neighbours without getting paid covers by no means the entire spectrum of prosocial behaviour. However, we feel that it constitutes a valid real-life approximation of a well-defined characteristic of prosocial behaviour. Observations on real-life human helping behaviour with friends and family basically approximates the degree of helpfulness a person exhibits in its everyday life. Unlike in standardized laboratory experiments we can only speculate on the reasons for these observations based on the information we have at hand (the questionnaire). Generally, helping behaviour towards friends and family may be accounted for by Hamilton’s rule of kin selection (family) or the basic principle of direct reciprocity [32]. The latter has often been targeted in well-constructed laboratory designs using (behavioural economic) settings in which participants interact–commonly under cover of anonymity–together in financially relevant interactions based on decisions on uncertainty. Trying to create an environment that resembles real-life interactions among fellow humans, interactions are being repeated over and over again, so that reputation and a history of (dis)trust can be established. From these studies we learned about facilitators and obstacles for the development of pro- and antisocial behaviour.

Using the data from the Health and Retirement Study we are able to go beyond this question. We can actually assess a degree of helpfulness in real-life. This comes of course with the cost of not being able to reproduce the motivations underlying these decisions.

The study at hand is limited to investigate a very narrow spectrum of human prosocial behaviour–namely individual differences in helping behaviour towards family and friends. And although it is not able to give answers similar to standardized (laboratory) studies, its exploratory approach might very well show new directions in investigating human prosocial behaviour.

## Results

Based on the University of Michigan's Health and Retirement Study (HRS), an on-going longitudinal panel study that collects survey data, anthropometric measurements, and physical performance tests, where more than 10,000 Americans have been genotyped, we used self-reported helping behaviour (SHB) to run a genetic association analysis on 1.2 Million SNPs.

One locus–rs11697300 –exceeded genome-wide significance in association with self-reported helping behaviour. Rs11697300 is an intergenic variant located between solute carrier family 52 (riboflavin transporter), member 3 (SLC52A3), and scratch family zinc finger 2 (SCRT2) on chromosome 20 (SNP = rs11697300, chromosome 20:718542, minor allele frequency (MAF) = 30.7%, *P* = 6.96 × 10^−10^). Table 1 lists the 10 SNPs with the lowest *P*-values.

**Table 1:** Summary of results of genetic association analyses.

SNP						GCTA-LOCO			PLINK		
Chr	Pos	ID	Ref	Alt	Freq	*b*	*s*.*e*.	*p*	*b*	*stat*	*p*
**20**	**718542**	**rs11697300**	**G**	**A**	**0.307**	**0.098**	**0.0159**	**6.96 × 10**^**−10**^	**0.1636**	**5.965**	**2.52 × 10**^**−9**^
4	42120509	rs2880666	G	A	0.377	-0.0863	0.0171	4.63 × 10^−7^	-0.1387	-4.661	3.19 × 10^−6^
4	42161427	rs6447133	G	A	0.242	-0.0889	0.0177	4.92 × 10^−7^	-0.1359	-4.436	9.24 × 10^−6^
4	42161491	rs6447134	G	A	0.235	-0.0892	0.0177	4.94 × 10^−7^	-0.1357	-4.415	1.02 × 10^−5^
4	42113241	rs13756	A	G	0.337	-0.0858	0.0172	5.82 × 10^−7^	-0.1378	-4.618	3.91 × 10^−6^
4	42074633	rs4619931	A	G	0.332	-0.0861	0.0174	7.24 × 10^−7^	-0.1361	-4.503	6.77 × 10^−6^
4	42112734	rs7682049	A	G	0.336	-0.0859	0.0172	8.07 × 10^−7^	-0.1374	-4.581	4.68 × 10^−6^
4	42089177	rs11051	G	A	0.333	-0.0856	0.0174	9.15 × 10^−7^	-0.1348	-4.442	9.02 × 10^−6^
4	42066378	rs10938175	A	G	0.374	-0.0865	0.0178	1.17 × 10^−6^	-0.1407	-4.548	5.47 × 10^−6^
4	42033153	rs9291209	A	G	0.288	-0.0839	0.0175	1.66 × 10^−6^	-0.1347	-4.434	9.36 × 10^−6^

SNP: single nucleotide polymorphism, Chr: chromosome, Pos: genomic position, ID: SNP name, Ref: reference allele, Alt: alternative allele, Freq: reference allele frequency. GCTA-LOCO: mixed-linear model implemented with GCTA's leaving-one-chromosome-out method with regression coefficient (*b*), standard error (*s*.*e*.), and p-value (*p*). PLINK: linear regression implemented with PLINK association analysis and PCA eigenvectors as covariates with regression coefficient (*b*), t-statistic (*stat*), and p-value (*p*).

**Fig 1:**
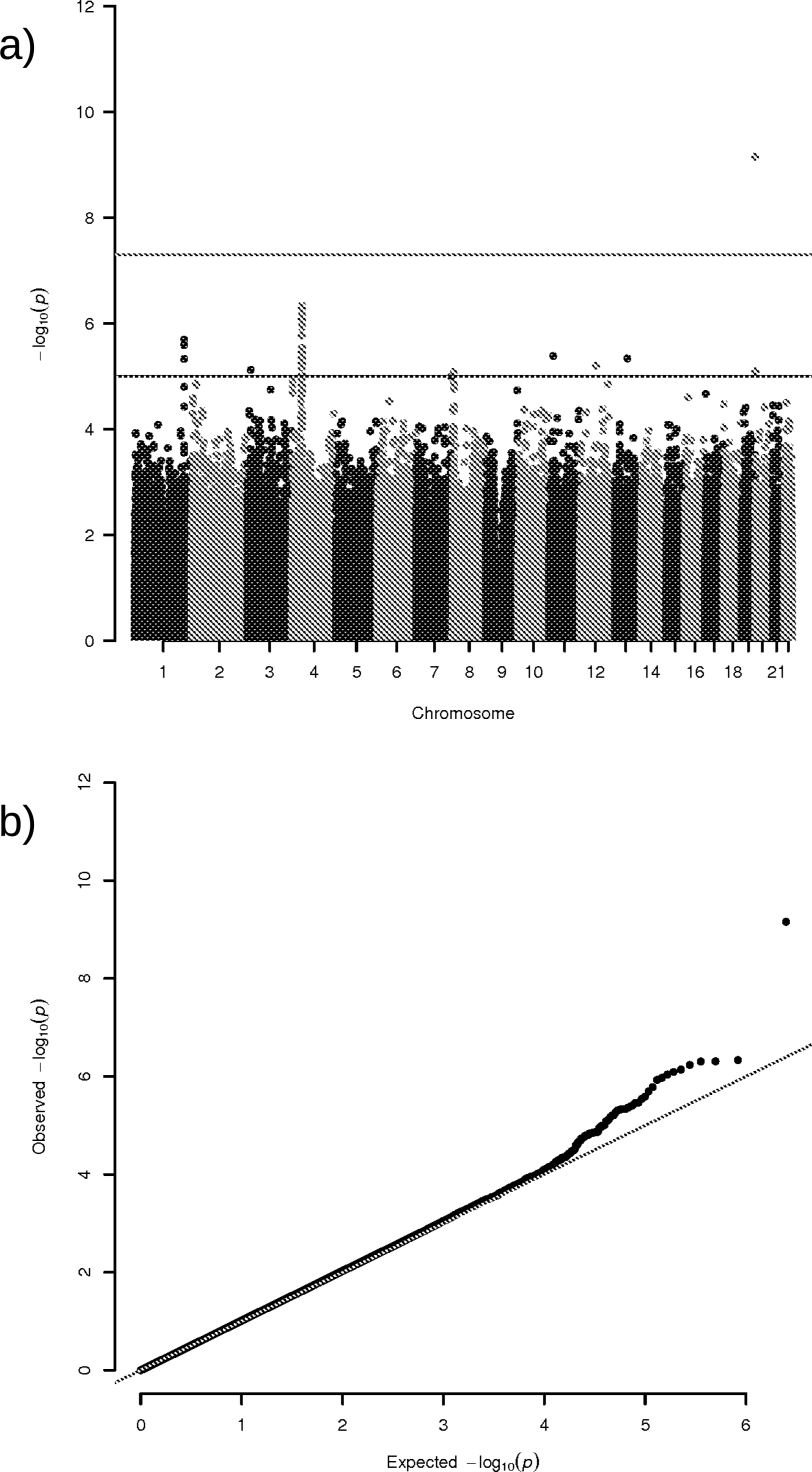
One locus on chromosome 20 reaches genome-wide significance in the GCTA-LOCO association analysis. (a) Manhattan plot of genome-wide association for self-reported helping behaviour. (b) Quantile-quantile plot of GWAS for self-reported helping behaviour.

Fig 1 shows Manhattan and Q-Q plots for association results.

Rs11697300 is located in a conserved region in the Hominidae. This is based on data from the UCSC Genome Browser (https://genome-euro.ucsc.edu/cgi-bin/hgTracks?db=hg38&lastVirtModeType=default&lastVirtModeExtraState=&virtModeType=default&virtMode=0&nonVirtPosition=&position=chr20%3A737890%2D737906&hgsid=225572689_6w3VFIL5xieR9y6lUUDNVbK6pABP). The Chimpanzee, the Orang—there is no data available for the Gorilla—as well as the phylogenetically closely related Gibbon show no differences in the region of interest.

Hence, rs11697300 seems to represent a phylogentic "old" variant in the Hominidae. However, drawing any further evolutionary conclusions on the basis on the available information must, at the moment, remain purely speculative.

**Fig 2:**
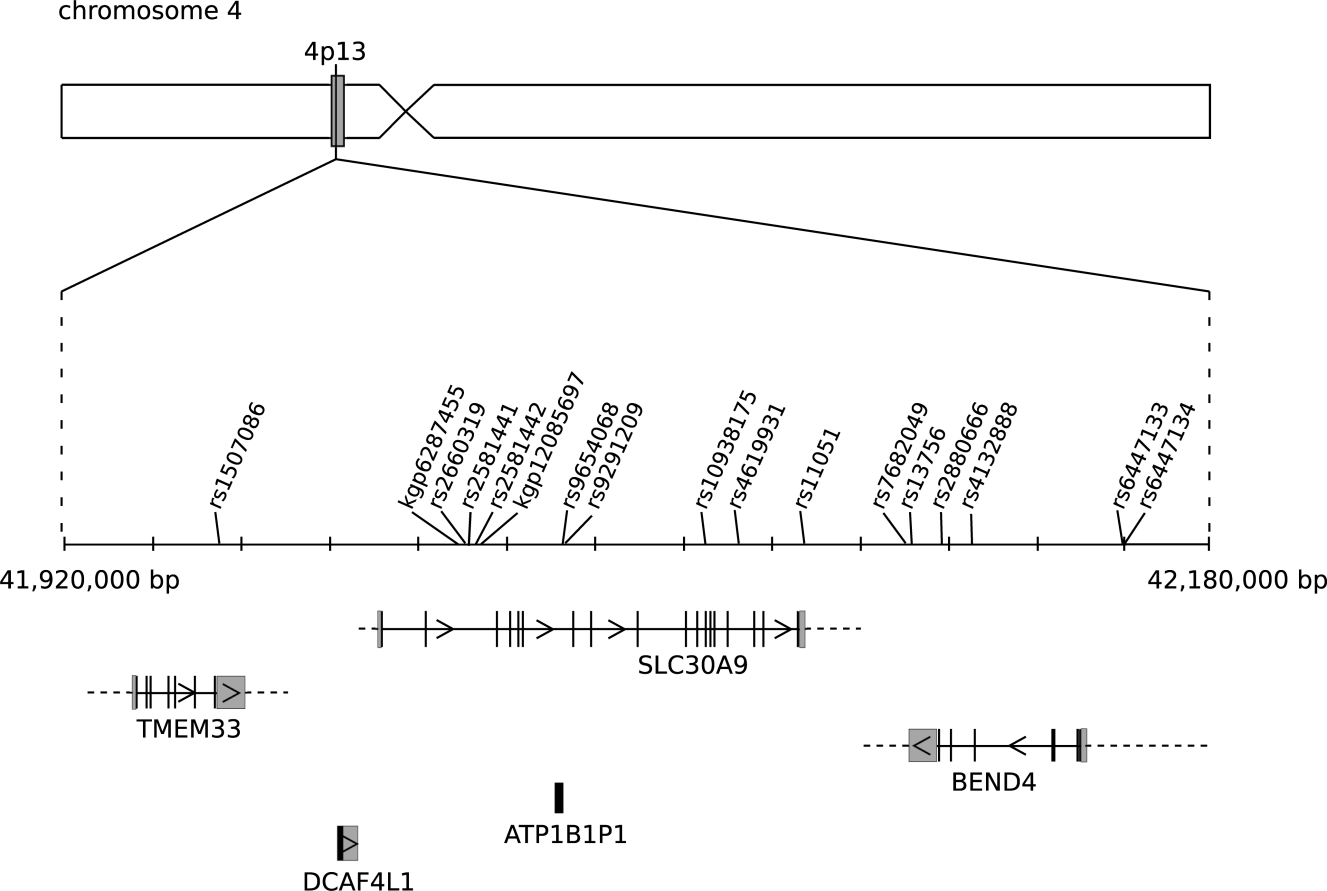
Schematic illustration of a region of 4p13. Within 215,932 base pairs, 17 single nucleotide polymorphisms (SNP) nearly reach genome-wide significance in association with self-reported helping behaviour. This area covers transmembrane protein 33 (TMEM33), DDB1 and CUL4 associated factor 4-like 1 (DCAF4L1), solute carrier family 30 (zinc transporter), member 9 (SLC30A9), ATPase, Na+/K+ transporting, beta 1 polypeptide pseudogene 1 (ATP1B1P1), and BEN domain containing 4 (BEND4). Black boxes depict exons, grey boxes are 5' and 3' untranslated regions.

Although only one locus reached genome-wide significance, association analysis revealed a striking pattern regarding a specific region on chromosome 4. The vast majority of SNPs approaching genome-wide significance (17 of 24 SNPs with *P* < 5 × 10^−6^) is located in a narrow region, spanning 215,932 base pairs, on chromosome 4 covering transmembrane protein 33 (TMEM33), DDB1 and CUL4 associated factor 4-like 1 (DCAF4L1), solute carrier family 30 (zinc transporter), member 9 (SLC30A9), ATPase, Na+/K+ transporting, beta 1 polypeptide pseudogene 1 (ATP1B1P1), and BEN domain containing 4 (BEND4) (Fig 2). S1 Table lists all SNPs with *P* values of association < 5 × 10^−6^.

We confirmed the robustness of the results of the genetic association analysis with a linear model including six covariates from principal component analysis (Methods, PLINK). Again, only one locus exceeded genome-wide significance in association with SHB (SNP = rs11697300, *P* = 2.52 × 10^−9^). And again, the area around SCL30A9 was revealed to be heavily populated with SNPs approaching genome-wide significance. Table 1 summarizes Top 10 SNPs for both genetic association analyses. S1 Fig shows Manhattan and Q-Q plots for PLINK results. Genomic inflation was estimated using the LD Score regression intercept to be 1.0318 (compare: λ_gc_ = 1.0466).

Genetic variance estimation was conducted following Yang et al. [33]). Using the GREML-LDMS method, we estimated from 10,713 unrelated individuals that 1,244,134 SNPs (MAF > 5%) explain 11% (standard error (s.e.) = 2.9%) of variance for self-reported helping behaviour (S2 Table).

Applying LDHub we found significant genetic correlations to the following GWAS: a) *New genetic loci implicated in fasting glucose homeostasis and their impact on type 2 diabetes risk*, by Dupuis et al. 2010 [34] (*P* = 0.0435); *b) Genome-wide study for circulating metabolites identifies 62 loci and reveals novel systemic effects of LPA*, by Kettunen et al. 2016 [35] (*P* = 0.0055); and c) *Genome-wide Association Studies Identify Genetic Loci Associated With Albuminuria in Diabetes*, by Teumer et al. 2016 [36] (*P* = 0.0313; *P* = 0.0434). Studies a) and b) are flagged as “Caution” by LDHub because “*using this data may yield results outside bounds due to relative low Z score*”. However, there seems to be a genetic correlation between the presented GWAS on SHB and GWAS on metabolism and diabetes (for a summary of the genetic correlations see Table 2).

**Table 2:** Genetic correlation estimates for SHB and selected traits.

	Trait	r_g_ (s.e.)	*P*-value
Top	Urinary albumin-to-creatinine ratio	-0.5739 (0.2665)	0.0313
	Urinary albumin-to-creatinine ratio (non-diabetes)	-0.5706 (0.2825)	0.0434
	Valine	-0.4362 (0.2518)	0.0833
	Fasting glucose main effect	-0.2220 (0.1321)	0.0925
	Infant head circumference	-0.3924 (0.2405)	0.1028
Null	Neuroticism	-0.2857 (0.2003)	0.1538
	Alzheimers disease	-0.2374 (0.2015)	0.2388
	Cognitive performance	0.1688 (0.1545)	0.2747
	Major depressive disorder	-0.1883 (0.2348)	0.4225
	Autism spectrum disorder	0.1080 (0.1424)	0.4483
	Schizophrenia	-0.0432 (0.0965)	0.6548

SHB: self-reported helping behaviour. The list of traits is grouped in to the top results (Top) and interesting null results (Null).

Gene set analysis revealed a total of 343 gene sets significantly associated (*P* < 0.05) with SNPs from the present SHB GWAS (S3 Table, S1 File 'gene-set-analysis.csv'), 26 of which with *P* < 10^−4^. Some of the gene sets found make biological sense, for instance gene sets involved in the synaptic membrane (*P* = 0.00004), dendritic (*P* = 0.00004) and neuron spine (*P* = 0.00004) and hormone receptor activity (*P* = 0.0007). Interestingly, some genes previously highlighted to influence prosocial behaviour are part of gene-sets significantly associated at *P* < 10^−4^: OXTR (adherents junction, telencephalon development), AVPR1a (telencephalon development), DRD4 (dendritic spine, neuron spine). Other interesting associated gene sets are negative regulation of behaviour (including DRD2, *P* = 0.004), learning (including COMT, DRD2, DRD3, DRD4, DRD5, *P* = 0.008), associative learning (including DRD1, DRD2, DRD3, DRD4, and DRD5, *P* = 0.041), and regulation of behaviour (including DRD1 and DRD2, *P* = 0.047).

## Discussion

Prosocial behaviour is a distinct human trait that is strongly influenced by genetic factors [6–8]. Our genome-wide association analysis was based on data collected by the Health and Retirement Study covering over 10,000 individuals and more than 1.2 million SNPs.

Our results indicate that one locus, rs11697300, an intergenic variant located between solute carrier family 52 (riboflavin transporter), member 3 (SLC52A3), and scratch family zinc finger 2 (SCRT2) on chromosome 20, is associated with self-reported helping behaviour. To date, no literature is available on the function of this variant or variants in strong linkage disequilibrium (LD) with rs11697300 (S4 Table, based on data provided by the 1000 Genomes Project [37], S5 Table, based on the HRS dataset providing P-values and effect sizes for all SNPs in high LD with rs11697300).

On chromosome 4, a pattern emerged revealing 17 variants approaching but not reaching genome-wide significance (S1 Table). All variants are located within 215,932 base pairs, an area containing the transmembrane protein 33 (TMEM33), DDB1 and CUL4 associated factor 4-like 1 (DCAF4L1), solute carrier family 30 (zinc transporter), member 9 (SLC30A9), ATPase, Na+/K+ transporting, beta 1 polypeptide pseudogene 1 (ATP1B1P1), and BEN domain containing 4 (BEND4) (Fig 2). None of these variants, however, have previously been described in the literature concerning functionality.

For the last decade, research concerned with genetic influences on prosocial behaviour focused on neuropeptides such as oxytocin and their pathway genes [38,39]. Our results suggest hint towards certain yet undescribed areas in the human genome to influence human helping behaviour. Note that, although we used two different methods to calculate the GWAS (GCTA and PLINK), we, due to the lack of comparable studies at hand, still miss the opportunity to replicate these results using a different data set to get more insights on the validity of the results provided by HRS data. Unfortunately, to our knowledge there is no other study available today that would qualify (either in scope or range of the study regarding the investigated behaviours) as a replication sample. Apart from that, this study is still subject to the general limitations common to all GWAS [40]: GWAS mainly report correlations between genetic loci and certain phenotypes. As a “correlational method“, a GWAS is unable to prove causality, as this is usually the case with correlational studies. A potential hint to the underlying biological mechanisms may be given by the genetic correlation and the gene set analysis we applied (discussed later). However, it will be necessary in future studies to investigate our results on a functional/physiological level, potentially clarifying the pathway from the genotype to the phenotype.

Moreover, due to the LD structure of the genome, GWAS are mainly designed to detect associations with relatively common variants in a population. Importantly, typical for GWA studies, the SNPs found to be significantly associated with a trait usually explain only a small proportion of the total variance. Accordingly, we applied the method of Yang et al. [29]–the estimation of the variance of a trait explained by all SNPs of a genome–to calculate the heritability due to additive effects of the trait “helping behaviour”. Due to the sample size of over 10k unrelated individuals this method yielded a robust estimate of heritability even for a substantially skewed measure of the trait “helping behaviour” (Table II)[41]. Existing studies on the heritability of prosocial behaviour report estimates between 10 and 60%. Estimates from 10 to 20% were found using a twin study design and cooperative behaviour in the trust game as a measure of behaviour [8]. 61% were found a twin study design by Knafo and Plomin 2006 [7] using parents and teacher ratings based on a validated behaviour questionnaire. While lower estimates are being achieved with measures of single behaviours (cooperative behaviour in the trust game), measures that combine observations of different behaviours [8] obtain a higher estimate. SHB presented in this study, yielding an estimate of 11%, however, only enabled measuring one dimension of human prosocial behaviour, namely “hours spent helping friends and family”. Therefore it is more comparable to the former method of measuring a single behaviour. We assume that additional data on prosocial behaviour which could be integrated into a more comprehensive variable on “prosocial behaviour” will become available in the future. Thus, bolstering the robustness of the measure might increase the “heritability coefficient” (the total variance explained by genome-wide data) according to the comprehensiveness of the measure in use.

However, our approach of heritability estimation is of course different from “classical” twin study designs to calculate heritability in prosocial behaviour (e.g. [7]) as the estimation of the variance of a trait is explained by all SNPs of a genome which are used to calculate the heritability due to additive effects of the trait s*elf-reported helping behaviour*.

Interestingly, albeit intuitively there no association between urinary albumin-to-creatinine ratio (microalbuminuria) would be expected, the genetic correlation between SHB and Albuminuria may make sense as Albuminuria is known of being associated with lower cognitive functioning particularly in elderly individuals [42,43]. If cognition in general is affected it could be speculated that prosocial behaviour may be affected as well. This may work directly by mutagenic or pleiotropic effects or indirectly via confounding effects of diseases. Comparable mechanisms may also hold true for the correlation of prosocial behaviour and lipoprotein blood levels, as there seems to be an association between cognition and lipoprotein blood levels [44]. However, at this stage such potential explanations for the genetic correlations must remain speculative, future studies far beyond the scope of this paper are needed.

Also the gene set analysis did find significant associations of the results to some gene sets that make biological sense including the dopamine receptor genes (DRD1 to DRD5), OXTR, and AVPR1a, all well known in the research of social behaviour. Especially associations with (associative) learning and (negative) regulation of behaviour appear intuitive and supportive of the results of the GWAS. However, as a “correlative approach” a GWAS is not able to transfer the vague concept of “genetic influence” in causality and determination. Accordingly, the relevance of the gene sets found to be associated with the results of the present GWAS may not be over-interpreted, but may provide a starting point for future analysis and deliver ideas where to start looking for causality and determination.

Based on our results we suggest that i) the potential function of rs11697300 and its surrounding area, as well as the other nearly genome-wide significant SNPs on and around SLC30A9, should be investigated in more detail; ii) rs11697300 and the other nearly genome-wide significant SNPs should be investigated in candidate-gene approaches, particularly in studies involving both laboratory-based experimental studies and studies on “every day” prosocial behaviour; iii) on the phenotypic level the accordance between lab and field data (laboratory-based experiments vs. “every day”prosocial behaviour) should be investigated in more detail because this issue is still under debate [25,45]; and iv) as mentioned above, additional GWA studies that sample a more comprehensive variety of “prosocial phenotypes” should be conducted in the future.

In conclusion, this study points towards new possible directions for research in behavioural genetics. We present results suggesting an association between yet undescribed genetic variants and human prosocial behaviour.

We encourage other studies to replicate and expand upon our findings. This would be an important step forward in clarifying the biological functioning of loci detected and supporting the notion that these areas are associated with prosocial behaviour.

## Material and methods

### Study description

The University of Michigan Health and Retirement Study (HRS) is an on-going longitudinal panel study designed to monitor changes in labour force participation and health transition of individuals toward the end of work life and beyond. The current sample population consists of 22,037 Americans over age 50. The sampling mechanism is based on a national probability sample to represent the entire American population. HRS collects survey data (demographic variables, physical and psychological well-being, life and job history, assets and financials, etc.), anthropometric measurements, and physical performance tests (e.g. body height, body weight, blood pressure, grip strength), as well as blood and saliva samples.

The Health and Retirement Study (Project #6192) genetic data is sponsored by the National Institute on Aging (grant numbers U01AG009740, RC2AG036495, and RC4AG039029) and was conducted by the University of Michigan [46]. Collection and production of HRS data comply with the requirements of the University of Michigan’s Institutional Review Board (IRB). For a detailed description of the study, see http://hrsonline.isr.umich.edu/index.php. This individual research project was approved by the Ethics Committee of the University of Vienna (Reference number 00077), data use was approved by the National Center for Biotechnology Information Genotypes and Phenotypes Database (NCBI dbGaP) Data Access Request system at the National Institutes of Health (Project ID 6192).

### Genotypic data

Based on voluntary participation, genotyping was performed on saliva samples. In total, 12,507 individuals have been genotyped since 2006. Genotyping was performed at the Center of Inherited Disease Research (CIDR) using the Illumina HumanOmni2.5-4v1 array and using the calling algorithm GenomeStudio version 2011.2, Genotyping Module 1.9.4 and GenTrain version 1.9. The medium call rate is 99.7% and the error estimated from 336 pairs of the study sample duplicates is 6 × 10^−5^. Further quality control steps were taken by teams at the University of Washington (UWGCC), the Health and Retirement Study investigator's team, and dbGaP. In total, 2,443,179 SNPs were genotyped. After several steps of stringent quality control measures, 1,244,134 SNPs were left for each participant Quality control steps included dropping dublicate SNPs and SNPs with a missing call rate > = 2%, Hardy-Weinberg-Equilibrium (HWE) P-value < 10^−4^ in either European or African samples, and a MAF < 0.05. Table 3 presents a detailed QC summary pipeline with the numbers of SNPs lost after each step (for more details on the process of quality control, see http://hrsonline.isr.umich.edu/sitedocs/genetics/HRS_QC_REPORT_MAR2012.pdf).

**Table 3:** Summary of quality control steps with the number of SNPs lost and kept for each step.

Filter	SNPs lost	SNPs kept
SNP probes		**2,443,179**
Genotyping failures	64,429	2,378,750
MAF = 0	60,705	2,318,045
Duplicate SNPs	10,162	2,307,883
Missing call rate > = 2%	89,017	2,218,866
> 4 discordant calls in 423 study duplicates	602	2,218,264
> 1 Mendelian error	1,450	2,216,814
HWE P-value < 10^−4^ in European or African samples	15,441	2,201,373
Sex difference in allelic frequency > = 0.2	2	2201371
% of SNPs lost due to quality control filters	9.90%	
MAF < 0.01	518989	1,682,382
% of SNPs lost due to qc filters + MAF filters	31.10%	
MAF < 0.05	957,237	**1,244,134**
% of SNPs lost due to qc filters + MAF filters	49.08%	

QC: quality control, SNP: single nucleotide polymorphism, MAF: minor allele frequency, HWE: Hardy-Weinberg-Equilibrium. Quality filters were applied sequentially in the order given.

After removing 172 related individuals (80 families of two and four families of three individuals) of the initial pool of 12,507 study participants 12,235 individuals were left in the subject pool. Families were defined as individuals being connected by a kinship coefficient (KC) > 0.1. The threshold corresponds to the expected KC of half-siblings minus two standard deviations.

### Phenotype

Self-reported helping behaviour (SHB) is coded in four questions (MG198, MG199, MG200, MG201) in section G (Functional Limitations and Helpers) of the Core questionnaire catalogue (The HRS 2010 Core Final Release (Version 5.0), public use dataset). The questions read as follows:

MG198, Have you spent any time in the past 12 months helping friends, neighbors, or relatives who did not live with you and did not pay you for the help? (1 = Yes, 5 = No)

MG199, Altogether, would you say the time amounted to less than 100 hours, more than 100 hours, or what? (1 = Less than 100, 3 = about 100, 5 = more than 100)

MG200, Would it be less than 200 hours, more than 200 hours, or what? (1 = Less than 200, 3 = about 200, 5 = more than 200)

MG201, Would it be less than 50 hours, more than 50 hours, or what? (1 = Less than 50, 3 = about 50, 5 = more than 50)

Based on these questions, we merged the eight possible combinations of answers into five categories of hours spent helping others: 0, 1 to 50, 51 to 100, 101 to 200, and 200+. Table 4 summarizes the possible combinations and gives the distribution of participants for each category.

**Table 4:** SHB is based on the answers of four questions on the amount of time an individual spent on helping others during the previous year.

SHB	MG198	MG199	MG200	MG201	Individuals
0	5	-	-	-	5288
1–50	1	1	-	1	3220
	1	1	-	3	
51–100	1	1	-	5	1177
	1	3	-	-	
101–200	1	5	1	-	579
	1	5	3	-	
200+	1	5	5	-	449
					10713

SHB: self-reported helping behaviour in hours spent previous year. MG198, MG199, MG200, and MG201 refer to four questions of section G (Functional Limitations and Helpers) of the 2010 Core questionnaire catalogue (Health and Retirement Study 2016). 1, 3, and 5 refer to categorical answers to respective questions. For details see main text. The column “individuals” refers to the total amount of participants allocated to respective SHB levels.

### Genetic association analysis

10,713 individuals with non-missing answers to SHB were matched to 1,244,134 SNPs. Genetic association analysis was carried out with i) a linear mixed model with a genetic relatedness matrix (GRM) and the effects of SNPs treated as random and ii) a standard linear regression approach with six principal component analysis eigenvectors as covariates.

#### GCTA

GCTA (version 1.25) provides options to perform mixed linear model (MLM)-based association analyses [47]. The MLM association technique is a widely recognized method of choice for association mapping when sample structure is present. It is based on constructing a GRM modelling the genome-wide sample structure. A random-effects model then estimates the contribution of the GRM to phenotypic variance, and association statistics are calculated to account for this phenotypic variance [48].

We implemented the GCTA-LOCO approach, which evaluates markers on a given chromosome using a GRM calculated from the remaining chromosomes. This 'leaving-one-chromosome-out' (LOCO) method avoids double-fitting the candidate marker and increases power of the analysis compared to regular MLM approaches as well as linear regression [48, 49].

#### PLINK

In PLINK (version 1.07) we used the implemented standard linear regression for quantitative trait data [50] to find potential associations of the genotype and self-reported helping behaviour, after including the eigenvectors of the PCA as covariates as recommended by the Health and Retirement Study for population stratification based on Patterson et al. [51]. PCA results are provided by the Health and Retirement Study. After LD pruning based on the set of autosomal SNPs with a missing call rate < 5%, MAF > 5%, and excluding the regions LCT, HLA, 8p23, and 17q21.31, 154,644 SNPs were selected for PCA. For details, see http://hrsonline.isr.umich.edu/sitedocs/genetics/HRS_QC_REPORT_MAR2012.pdf.

### Genomic inflation

We used the python tool LDSC to estimate genomic inflation (https://github.com/bulik/ldsc/wiki/Heritability-and-Genetic-Correlation). LDSC calculates genomic inflation as the proportion of the inflation in the mean χ2 that the LD Score regression attributes to causes other than polygenic heritability [52]. Using the LD Score regression intercept as an estimate of inflation, the estimate is, other than λ_gc_, not biased by sample size in the presence of polygenicity [53].

### Genetic variance estimation

We estimated genetic variance based on GCTA's GREML-LDMS method [33] using whole genome sequence data. As this method cannot account for variance attributable to extremely rare causal variant or variants that are not polymorphic in the dataset, we calculated a slight underestimate of the genetic variance. The analysis is conducted in four steps using GCTA [47] (steps i, iii, and iv) and R statistical programming software [54] (step ii). The first step is to calculate the segment-based LD score (i). Subsequently, SNP stratification (ii) is done based on (i) and MAF. Stratified SNPs are used to calculate four GRMs based on the quartiles of the ld score (iii), which are then used as multiple GRMs in performing a REML analysis (iv) [33].

### Genetic correlation

We used the online tool LDHub (http://ldsc.broadinstitute.org) to estimate potential genetic correlations among SHB and 177 diseases and traits gathered from publicly available resources and consortia. Estimation is done on the basis of the summary level results of the present GWAS on SHB and the summary results of those 177 GWAS [55].

LDhub has been implemented on basis of Bulik-Sullivan et al. 2015a [52], Bulik-Sullivan et al. 2015b [31]. This method regresses the summary results statistics of GWAS including the genetic variants across the genome measuring each variant’s ability to tag other variants locally (detailed explanation can be found in Bulik-Sullivan et al. 2015a [51]).

### Gene set analysis

We applied the gene set analysis (GSA) approach developed by Nam et al [30] implementing in the Java application “GSA SNP” (https://sourceforge.net/projects/gsa-snp/files/?source=navbar) on the present GWAS results (SNP with its *P* value from the GWAS). GSA assigns SNPs to a gene that encompasses the SNP with some padding. Genes are clustered in gene sets of known function. As gene set we used the set “Gene Ontology” (default) with a padding size of +/- 20,000 and k-th best *P* value (default 2). *P* values are corrected according to Benjamini and Hochberg [56]. The GSA-SNP analysis uses the PAGE method [57]. Details to the method can be found in Nam et al. 2010 [30] and Kim et al. 2005 [56].

## Supporting information

S1 Fig One locus on chromosome 20 reaches genome-wide significance in the PLINK association analysis. (a) Manhattan plot of genome-wide association for self-reported helping behaviour. (b) Quantile-quantile plot of GWAS for self-reported helping behaviour.
(EPS)

S1 File Gene-set analysis. Gene set analysis revealed a total of 343 gene sets significantly associated (*P* < 0.05) with SNPs. Information includes set name, gene count, set size, z-score, p-value, corrected p-value, FDR, and gene symbols.
(CSV)

S1 Table Summary results of genetic association analyses for SNPs with *P* values < 5 x 10^−6^ (GCTA-LOCO). SNP: Single nucleotide polymorphism, Chr: chromosome, Pos: base pair position, ID: SNP name, Ref: reference allele, Alt: alternative allele, Freq: reference allele frequency. GCTA-LOCO: mixed-linear model implemented with GCTA's leaving-one-chromosome-out method with regression coefficient (*b*), standard error (*se*), and p-value (*p*). PLINK: linear regression implemented with PLINK association analysis and PCA eigenvectors as covariates with regression coefficient (*b*), t-statistic (*stat*), and p-value (*p*). 17 SNPs located within 215,932 base pairs on chromosome 4 are highlighted in bold.
(DOCX)

S2 Table Estimates of variance explained from GREML-LDMS analysis for self-reported helping behaviour. GREML-LDMS (Yang et al. 2015): Linkage disequilibrium and minor allele frequency stratified GREML analysis with estimates (*Est*) and standard errors (*s*.*e*.); for details see main text.
(DOCX)

S3 Table A selection of gene sets strongly associated with SHB. SHB: self-reported helping behaviour. The list of gene sets is grouped in to the top results (Top) and interesting results (Misc).
(DOCX)

S4 Table SNPs in strong linkage disequilibrium (LD) with rs11697300 based on data provided by the 1000 Genomes Project (Consortium 2012). SNP: single nucleotide polymorphism. Data available on http://www.ensembl.org/Homo_sapiens/Variation/Explore?db=core;r=20:737398-738398;v=rs11697300;vdb=variation;vf=107862839. ASW: African Ancestry in Southwest US, CEU: Utah residents with Northern and Western European Ancestry, MXL: Mexican Ancestry in Los Angeles, California. Populations were chosen to represent the Health and Retirement Study sample population.
(DOCX)

S5 Table SNPs in strong linkage disequilibrium (LD) with rs11697300 based on the HRS dataset. SNP: single nucleotide polymorphism, Chr: chromosome, Pos: genomic position, ID: SNP name, Ref: reference allele, Alt: alternative allele, Freq: reference allele frequency, r^2^: LD score with rs11697300. GCTA-LOCO: mixed-linear model implemented with GCTA's leaving-one-chromosome-out method with regression coefficient (*b*), standard error (*s*.*e*.), and p-value (*p*).
(DOCX)
