# Supplementary material for: Real-life helping behaviours in North America: A genome-wide association approach
Source: PLoS One. 2018 Jan 11;13(1):e0190950. doi: 10.1371/journal.pone.0190950 (PMC5764334; doi:10.1371/journal.pone.0190950)
Supplement: S1 Table — SNP: Single nucleotide polymorphism, Chr: chromosome, Pos: base pair position, ID: SNP name, Ref: reference allele, Alt: alternative allele, Freq: reference allele frequency. GCTA-LOCO: mixed-linear model implemented with GCTA's leaving-one-chromosome-out method with regression coefficient (b), standard error (se), and p-value (p). PLINK: linear regression implemented with PLINK association analysis and PCA eigenvectors as covariates with regression coefficient (b), t-statistic (stat), and p-value (p). 17 SNPs located within 215,932 base pairs on chromosome 4 are highlighted in bold. (DOCX) [file pone.0190950.s003.docx]

**S1 Table. Summary results of genetic association analyses for SNPs with *P* values < 5  10^-6^** **(GCTA-LOCO).**

| **SNP** | | | | | | |  | | **GCTA-LOCO** | | | |  | | **PLINK** | | |
| --- | --- | --- | --- | --- | --- | --- | --- | --- | --- | --- | --- | --- | --- | --- | --- | --- | --- |
| Chr | Pos | ID | Ref | Alt | Freq |  | | *b* | | *s.e.* | *p* |  | | *b* | | *stat* | *p* |
| 20 | 718542 | rs11697300 | G | A | 0.307 |  | | 0.098 | | 0.0159 | 6.96 × 10^-10^ |  | | 0.1636 | | 5.965 | 2.52 × 10^-9^ |
| **4** | **42120509** | **rs2880666** | **G** | **A** | **0.377** |  | | **-0.0863** | | **0.0171** | **4.63 × 10^-7^** |  | | **-0.1387** | | **-4.661** | **3.19 × 10^-6^** |
| **4** | **42161427** | **rs6447133** | **G** | **A** | **0.242** |  | | **-0.0889** | | **0.0177** | **4.92 × 1^-7^** |  | | **-0.1359** | | **-4.436** | **9.24 × 10^-6^** |
| **4** | **42161491** | **rs6447134** | **G** | **A** | **0.235** |  | | **-0.0892** | | **0.0177** | **4.94 × 10^-7^** |  | | **-0.1357** | | **-4.415** | **1.02 × 10^-5^** |
| **4** | **42113241** | **rs13756** | **A** | **G** | **0.337** |  | | **-0.0858** | | **0.0172** | **5.82 × 10^-7^** |  | | **-0.1378** | | **-4.618** | **3.91 × 10^-6^** |
| **4** | **42074633** | **rs4619931** | **A** | **G** | **0.332** |  | | **-0.0861** | | **0.0174** | **7.24 × 10^-7^** |  | | **-0.1361** | | **-4.503** | **6.77 × 10^-6^** |
| **4** | **42112734** | **rs7682049** | **A** | **G** | **0.336** |  | | **-0.0859** | | **0.0172** | **8.07 × 10^-7^** |  | | **-0.1374** | | **-4.581** | **4.68 × 10^-6^** |
| **4** | **42089177** | **rs11051** | **G** | **A** | **0.333** |  | | **-0.0856** | | **0.0174** | **9.15 × 10^-7^** |  | | **-0.1348** | | **-4.442** | **9.02 × 10^-6^** |
| 0 | 0 | kgp22787791 | C | A | 0.328 |  | | -0.0861 | | 0.0176 | 1.07 × 10^-6^ |  | | -0.1391 | | -4.624 | 3.81 × 10^-6^ |
| **4** | **42066378** | **rs10938175** | **A** | **G** | **0.374** |  | | **-0.0865** | | **0.0178** | **1.17 × 10^-6^** |  | | **-0.1407** | | **-4.548** | **5.47 × 10^-6^** |
| **4** | **42033153** | **rs9291209** | **A** | **G** | **0.288** |  | | **-0.0839** | | **0.0175** | **1.66 × 10^-6^** |  | | **-0.1347** | | **-4.434** | **9.36 × 10^-6^** |
| 1 | 216566778 | rs6664755 | G | A | 0.341 |  | | -0.0759 | | 0.0159 | 2.02 × 10^-6^ |  | | -0.1253 | | -4.534 | 1.66 × 10^-6^ |
| 1 | 216549828 | kgp8154610 | G | A | 0.392 |  | | -0.0728 | | 0.0155 | 2.58 × 10^-6^ |  | | -0.1257 | | -4.696 | 1.66 × 10^-6^ |
| **4** | **42125621** | **rs4132888** | **G** | **A** | **0.341** |  | | **-0.0805** | | **0.0172** | **2.89 × 10^-6^** |  | | **-0.1305** | | **-4.356** | **1.34 × 10^-5^** |
| **4** | **41956413** | **rs1507086** | **G** | **A** | **0.335** |  | | **-0.0802** | | **0.0173** | **3.41 × 10^-6^** |  | | **-0.1280** | | **-4.259** | **2.07 × 10^-5^** |
| **4** | **42011813** | **rs2581442** | **A** | **G** | **0.275** |  | | **-0.0805** | | **0.0173** | **3.46 × 10^-6^** |  | | **-0.1318** | | **-4.386** | **1.17 × 10^-5^** |
| **4** | **42009839** | **kgp6287455** | **A** | **G** | **0.288** |  | | **-0.0808** | | **0.0175** | **3.88 × 10^-6^** |  | | **-0.1307** | | **-4.307** | **1.67 × 10^-5^** |
| 11 | 18257123 | rs2925145 | A | C | 0.554 |  | | 0.0683 | | 0.0148 | 4.11 × 10^-6^ |  | | 0.1078 | | 4.198 | 2.71 × 10^-5^ |
| **4** | **42010884** | **rs2660319** | **G** | **A** | **0.312** |  | | **-0.0797** | | **0.0172** | **4.38 × 10^-6^** |  | | **-0.1283** | | **-4.287** | **1.83 × 10^-5^** |
| 13 | 18257123 | rs7984233 | A | G | 0.250 |  | | 0.0782 | | 0.0171 | 4.59 × 10^-6^ |  | | 0.1259 | | 4.269 | 1.98 × 10^-6^ |
| **4** | **42010884** | **rs2581441** | **A** | **G** | **0.319** |  | | **-0.0797** | | **0.0174** | **4.63 × 10^-6^** |  | | **-0.1278** | | **-4.287** | **2.38 × 10^-5^** |
| 1 | 216552091 | rs2669053 | G | A | 0.391 |  | | -0.0710 | | 0.0155 | 4.67 × 10^-6^ |  | | -0.1224 | | -4.562 | 5.13 × 10^-6^ |
| **4** | **42014071** | **kgp12085697** | **C** | **A** | **0.287** |  | | **-0.0800** | | **0.0175** | **4.79 × 10^-6^** |  | | **-0-1301** | | **-4-284** | **1.85 × 10^-5^** |
| **4** | **42032966** | **rs9654068** | **G** | **A** | **0.288** |  | | **-0.0799** | | **0.0175** | **4.99 × 10^-6^** |  | | **-0.1308** | | **-4.306** | **1.68 × 10^-5^** |
| SNP: Single nucleotide polymorphism, Chr: chromosome, Pos: base pair position, ID: SNP name, Ref: reference allele, Alt: alternative allele, Freq: reference allele frequency. GCTA-LOCO: mixed-linear model implemented with GCTA's leaving-one-chromosome-out method with regression coefficient (*b*), standard error (*se*), and p-value (*p*). PLINK: linear regression implemented with PLINK association analysis and PCA eigenvectors as covariates with regression coefficient (*b*), t-statistic (*stat*), and p-value (*p*). 17 SNPs located within 215,932 base pairs on chromosome 4 are highlighted in bold. | | | | | | | | | | | | | | | | | |
