# Supplementary material for: Real-life helping behaviours in North America: A genome-wide association approach
Source: PLoS One. 2018 Jan 11;13(1):e0190950. doi: 10.1371/journal.pone.0190950 (PMC5764334; doi:10.1371/journal.pone.0190950)
Supplement: S2 Table — GREML-LDMS (Yang et al. 2015): Linkage disequilibrium and minor allele frequency stratified GREML analysis with estimates (Est) and standard errors (s.e.); for details see main text. (DOCX) [file pone.0190950.s004.docx]

**S2 Table****. Estimates of variance explained from GREML-LDMS analysis for self-reported helping behaviour.**

|  | 1^st^ LD quartile | | 2^nd^ LD quartile | | | | 3^rd^ LD quartile | | | | 4^th^ LD quartile | | | | |
| --- | --- | --- | --- | --- | --- | --- | --- | --- | --- | --- | --- | --- | --- | --- | --- |
|  | *Est* | *s.e.* | | *Est* | | *s.e.* | | | *Est* | *s.e.* | | | *Est* | | *s.e.* |
| SHB | 0.0121 | 0.0256 | | 0.0735 | | 0.0276 | | | 0.0196 | 0.0267 | | | 0.0241 | | 0.0214 |
|  |  |  | |  | |  | | |  |  | | |  | |  |
| Total sum | 0.1111 | 0.0294 | |  | |  | | |  |  | | |  | |  |
| Log likelihood | -6,149,926 | |  | |  | | |  | |  | |  | |  | |
| Sample size | 10,713 |  | |  | |  | | |  |  | | |  | |  |
| GREML-LDMS (Yang et al. 2015): Linkage disequilibrium and minor allele frequency stratified GREML analysis with estimates (*Est*) and standard errors (*s.e.*); for details see main text. | | | | | | | | | | | | | | | |
