# Supplementary material for: Real-life helping behaviours in North America: A genome-wide association approach
Source: PLoS One. 2018 Jan 11;13(1):e0190950. doi: 10.1371/journal.pone.0190950 (PMC5764334; doi:10.1371/journal.pone.0190950)
Supplement: S3 Table — SHB: self-reported helping behaviour. The list of gene sets is grouped in to the top results (Top) and interesting results (Misc). (DOCX) [file pone.0190950.s005.docx]

**S3 Table. A selection of gene sets strongly associated with SHB.**

|  | **gene set** | **gene count** | ***P*-value** |
| --- | --- | --- | --- |
| Top | transmembrane receptor protein kinase activity | 82 | 0.000001 |
|  | extracellular matrix part | 177 | 0.000001 |
|  | transmembrane receptor protein tyrosine kinase activity | 65 | 0.000001 |
|  | homophilic cell adhesion | 94 | 0.000003 |
|  | synaptic membrane | 186 | 0.000043 |
|  | dendritic spine | 144 | 0.000043 |
|  | neuron spine | 144 | 0.000043 |
|  | calcium ion transmembrane transporter activity | 104 | 0.000193 |
| Misc | steroid hormone receptor activity | 48 | 0.000751 |
|  | negative regulation of behavior | 26 | 0.004379 |
|  | learning | 81 | 0.008343 |
|  | associative learning | 48 | 0.040726 |
|  | regulation of behavior | 125 | 0.046641 |
| SHB: self-reported helping behavior. The list of gene sets is grouped in to the top results (Top) and interesting results (Misc). | | | |
