# Supplementary material for: Real-life helping behaviours in North America: A genome-wide association approach
Source: PLoS One. 2018 Jan 11;13(1):e0190950. doi: 10.1371/journal.pone.0190950 (PMC5764334; doi:10.1371/journal.pone.0190950)
Supplement: S4 Table — SNP: single nucleotide polymorphism. Data available on http://www.ensembl.org/Homo_sapiens/Variation/Explore?db=core;r=20:737398-738398;v=rs11697300;vdb=variation;vf=107862839. ASW: African Ancestry in Southwest US, CEU: Utah residents with Northern and Western European Ancestry, MXL: Mexican Ancestry in Los Angeles, California. Populations were chosen to represent the Health and Retirement Study sample population. (DOCX) [file pone.0190950.s006.docx]

**S4 Table. SNPs in strong linkage disequilibrium (LD) with rs11697300 based on data provided by the 1000 Genomes Project** **(Consortium 2012)**.

|  | ASW | | CEU | | MXL | |
| --- | --- | --- | --- | --- | --- | --- |
| SNP | r² | D' | r² | D' | r² | D' |
| rs112465128 |  |  |  | 0.999 |  | 0.999 |
| rs1040739 |  | 1 |  | 0.824 |  |  |
| rs114141496 |  | 0.889 |  |  |  |  |
| rs114168816 |  | 1 |  |  |  |  |
| rs115178198 |  | 1 |  |  |  |  |
| rs115975747 |  | 1 |  |  |  |  |
| rs116686887 |  | 1 |  |  |  |  |
| rs12624604 |  |  |  |  |  | 0.878 |
| rs139427693 |  |  |  |  |  | 0.904 |
| rs142981876 |  |  |  |  |  | 0.904 |
| rs146563696 |  | 1 |  |  |  |  |
| rs149498177 |  | 1 |  |  |  |  |
| rs17562054 |  |  |  |  |  | 0.904 |
| rs1884639 |  |  |  | 1 |  | 1 |
| rs2016390 |  | 0.949 |  |  |  | 1 |
| rs2093013 | 0.921 | 0.96 |  | 0.973 | 0.934 | 1 |
| rs2317021 |  | 1 |  | 1 |  | 0.955 |
| rs2317022 |  | 1 |  | 1 |  | 0.955 |
| rs2422518 |  | 1 |  | 1 |  |  |
| rs2422519 |  |  |  | 1 |  |  |
| rs2873339 |  | 1 |  | 0.884 |  |  |
| rs4263183 |  |  |  |  |  | 1 |
| rs4386392 |  |  |  |  |  | 1 |
| rs4387876 |  |  |  |  |  | 1 |
| rs4572665 |  |  |  |  |  | 1 |
| rs4577350 |  |  |  |  |  | 1 |
| rs538132895 |  | 1 |  |  |  |  |
| rs55781505 |  |  |  |  |  | 0.801 |
| rs56411166 |  |  |  | 0.822 |  |  |
| rs56815619 |  |  |  | 1 |  | 0.94 |
| rs56839494 |  |  |  | 1 |  | 1 |
| rs570271046 |  |  |  |  |  |  |
| rs58674686 |  |  |  | 1 |  | 0.94 |
| rs59488666 |  |  |  |  |  | 1 |
| rs6038545 |  |  |  | 1 |  |  |
| rs6038592 |  | 1 |  |  |  |  |
| rs6054380 |  | 0.996 |  | 1 |  | 1 |
| rs6054423 |  | 1 |  | 1 |  | 0.955 |
| rs6054497 |  |  |  | 1 |  | 1 |
| rs6077036 |  |  |  | 0.937 |  |  |
| rs6085641 |  | 1 |  | 0.827 |  |  |
| rs6085645 |  | 1 |  | 1 |  | 1 |
| rs60957781 |  |  |  |  |  | 1 |
| rs6107838 |  |  |  |  |  | 0.815 |
| rs6107859 |  | 1 |  |  |  |  |
| rs6107866 |  |  |  | 0.927 |  | 1 |
| rs6117337 |  |  |  | 1 |  | 0.819 |
| rs6117338 |  |  |  | 0.876 |  |  |
| rs6117340 |  |  |  | 0.935 |  |  |
| rs6117349 |  |  |  |  |  | 0.815 |
| rs6117372 |  |  |  | 1 |  |  |
| rs6117373 |  |  |  | 1 |  |  |
| rs6117374 |  | 1 |  | 1 |  | 1 |
| rs6117402 |  |  |  |  |  | 0.873 |
| rs6133337 |  | 1 |  | 1 |  | 0.955 |
| rs6133340 |  | 1 |  | 0.824 |  |  |
| rs6133341 |  | 0.949 |  |  |  | 1 |
| rs6140037 |  |  |  |  |  | 0.904 |
| rs6140042 |  | 1 |  |  |  | 1 |
| rs6140045 |  | 1 |  | 0.824 |  |  |
| rs6140046 |  | 0.949 |  |  |  | 1 |
| rs6140047 | 0.921 | 0.96 | 0.803 | 0.973 | 0.934 | 1 |
| rs6140053 |  | 1 |  |  |  | 1 |
| rs6140073 |  | 1 |  |  |  |  |
| rs61489106 |  |  |  | 0.955 |  |  |
| rs67843837 |  |  |  | 0.882 |  |  |
| rs7268129 |  | 1 |  |  |  | 1 |
| rs74181555 |  | 0.829 |  |  |  |  |
| rs74834673 |  | 1 |  |  |  |  |
| rs75235412 |  | 1 |  |  |  |  |
| rs75987662 |  | 1 |  |  |  |  |
| rs76740411 |  | 1 |  |  |  |  |
| rs77115330 |  |  |  | 1 |  | 1 |
| rs78004369 |  |  |  |  |  | 1 |
| rs79533028 |  | 1 |  |  |  |  |
| rs80031595 |  | 1 |  | 1 |  | 1 |
| rs80054732 |  |  |  |  |  | 1 |
| rs80269253 |  |  |  |  |  | 0.904 |
| rs80329228 |  |  |  |  |  | 1 |
| rs927067 |  | 1 |  | 1 |  | 1 |
| rs975449 |  | 0.94 |  | 1 |  | 0.959 |
| rs9789781 |  |  |  |  |  | 1 |
| rs9789826 |  |  |  |  |  | 1 |
| rs9789828 |  |  |  |  |  | 1 |
| rs9789832 |  |  |  |  |  | 1 |
| rs9789833 |  |  |  |  |  | 1 |
| SNP: single nucleotide polymorphism. Data available on <http://www.ensembl.org/Homo_sapiens/Variation/Explore?db=core;r=20:737398-738398;v=rs11697300;vdb=variation;vf=107862839>. ASW: African Ancestry in Southwest US, CEU: Utah residents with Northern and Western European Ancestry, MXL: Mexican Ancestry in Los Angeles, California. Populations were chosen to represent the Health and Retirement Study sample population. | | | | | | |
