# Supplementary material for: Real-life helping behaviours in North America: A genome-wide association approach
Source: PLoS One. 2018 Jan 11;13(1):e0190950. doi: 10.1371/journal.pone.0190950 (PMC5764334; doi:10.1371/journal.pone.0190950)
Supplement: S5 Table — SNP: single nucleotide polymorphism, Chr: chromosome, Pos: genomic position, ID: SNP name, Ref: reference allele, Alt: alternative allele, Freq: reference allele frequency, r2: LD score with rs11697300. GCTA-LOCO: mixed-linear model implemented with GCTA's leaving-one-chromosome-out method with regression coefficient (b), standard error (s.e.), and p-value (p). (DOCX) [file pone.0190950.s007.docx]

**S5 Table. SNPs in strong linkage disequilibrium (LD) with rs11697300 based on the** **HRS dataset**.

| **SNP** | | | | | | |  | **GCTA-LOCO** | | |
| --- | --- | --- | --- | --- | --- | --- | --- | --- | --- | --- |
| Chr | Pos | ID | Ref | Alt | Freq | r^2^ |  | *b* | *s.e.* | *p* |
| 20 | 718542 | rs11697300 | G | A | 0.31 | 1 |  | 0.0980 | 0.0159 | 6.96 × 10^-10^ |
| 20 | 715571 | rs2016390 | A | G | 0.18 | 0.67 |  | 0.0667 | 0.0190 | 0.0005 |
| 20 | 714261 | rs975449 | A | G | 0.48 | 0.53 |  | 0.0467 | 0.0148 | 0.0016 |
| 20 | 736861 | rs11905326 | A | G | 0.31 | 0.48 |  | 0.0372 | 0.0162 | 0.0212 |
| 20 | 737052 | rs7351978 | A | G | 0.31 | 0.48 |  | 0.0387 | 0.0161 | 0.0163 |
| 20 | 722733 | rs77115330 | A | C | 0.09 | 0.44 |  | 0.0573 | 0.0254 | 0.0244 |
| 20 | 733097 | rs57285046 | A | G | 0.09 | 0.43 |  | 0.0388 | 0.0256 | 0.1293 |
| 17 | 4883818 | rs238234 | C | G | 0.17 | 0.42 |  | 0.0551 | 0.0196 | 0.0050 |
| 16 | 13267393 | rs2550566 | C | A | 0.19 | 0.42 |  | -0.0114 | 0.0189 | 0.5450 |
| 8 | 128571759 | rs4313118 | G | A | 0.2 | 0.41 |  | 0.0298 | 0.0184 | 0.1058 |
| 1 | 183247090 | rs10494561 | A | G | 0.11 | 0.41 |  | 0.0021 | 0.0235 | 0.9299 |
| 1 | 183241781 | rs16860727 | A | G | 0.11 | 0.41 |  | 0.0082 | 0.0232 | 0.7243 |
| 8 | 28092874 | rs36015978 | G | C | 0.13 | 0.40 |  | -0.0157 | 0.2197 | 0.4762 |
| SNP: single nucleotide polymorphism, Chr: chromosome, Pos: genomic position, ID: SNP name, Ref: reference allele, Alt: alternative allele, Freq: reference allele frequency, r^2^: LD score with rs11697300. GCTA-LOCO: mixed-linear model implemented with GCTA's leaving-one-chromosome-out method with regression coefficient (*b*), standard error (*s.e.*), and p-value (*p*). | | | | | | | | | | |
